# Transcriptomic Interaction between Young Fecal Transplantation and Perfluorobutanesulfonate in Aged Zebrafish Gonads

**DOI:** 10.3390/toxics10110631

**Published:** 2022-10-22

**Authors:** Lizhu Tang, Jing Li, Baili Sun, Yachen Bai, Xiangzhen Zhou, Lianguo Chen

**Affiliations:** 1 State Key Laboratory of Freshwater Ecology and Biotechnology, Institute of Hydrobiology, Chinese Academy of Sciences, Wuhan 430072, China; 2 University of Chinese Academy of Sciences, Beijing 100049, China

**Keywords:** aging, PFBS, young fecal transplantation, transcriptome, gonad

## Abstract

The transfer of young fecal microbiota has been found to significantly refresh the reproductive endocrine system and effectively ameliorate the toxicity of perfluorobutanesulfonate (PFBS) in aged zebrafish recipients. However, the mechanisms underlying the antagonistic action of young fecal microbiota against the reproductive endocrine toxicity of PFBS remain largely unknown. In this study, the aged zebrafish were transplanted with feces from young donors and then exposed to PFBS for 14 days. After exposure, the shift in the transcriptomic fingerprint of the gonads was profiled by using high-throughput sequencing, aiming to provide mechanistic clues into the interactive mode of action between young fecal transplantation and PFBS’s innate toxicity. The results showed that the gene transcription pattern associated with protein and lipid synthesis in the gonads of the aged individuals was quite different from the young counterparts. It was intriguing that the transplantation of young feces established a youth-like transcriptomic phenotype in the elderly recipients, thus attenuating the functional decline and maintaining a healthy aging state of the gonads. A sex specificity response was clearly observed. Compared to the aged females, more metabolic pathways (e.g., glycine, serine, and threonine metabolism; glyoxylate and dicarboxylate metabolism; pyrimidine metabolism) were significantly enriched in aged males receiving young feces transplants. PFBS dramatically altered the transcriptome of aged testes, while a much milder effect was observable in aged ovaries. Accordingly, a suite of biological processes related to germ cell proliferation were disrupted by PFBS in aged males, including the ECM–receptor interaction, retinol metabolism, and folate biosynthesis. In aged ovaries exposed to PFBS, mainly the fatty acid and arginine biosynthesis pathway was significantly affected. However, these transcriptomic disorders caused by PFBS were largely mitigated in aged gonads by transferring young feces. Overall, the present findings highlighted the potential of young fecal transplantation to prevent the functional compromise of gonads resulting from aging and PFBS.

## 1. Introduction

Aging implies biological tissue damage and degeneration, defective self-repair, and susceptibility to aging-related diseases, which may eventually lead to the declining viability of the animals [1]. Aging-triggered changes in the organism are also accompanied by the transition in the intrinsic gut microbial flora. It has been recognized that gut microbiota gradually evolves alongside aging progression [2]. Aged gut microbiota is generally characterized by dramatic fluctuations in the abundance, diversity, and metabolism of intestinal microbes compared to young individuals, leading to pathogen enrichment [3]. The importance of the gut microbial ecosystem is increasingly appreciated, as it maintains host health by regulating a battery of physiological activities such as energy metabolism, immune response, and neural signaling [4,5]. Therefore, the intestinal microbial dysfunction resulting from aging can subsequently increase the intestinal permeability, which is a major cause of chronic low-degree systemic inflammation and premature death of the host animals [6]. There is a growing body of evidence that confirms the interplay between aging and gut microbes; that is, while aging has physiological effects on the resident microbiota, host–microbiota interactions similarly affect the rate and extent of aging [7]. As a central component to many age-related changes, the gut microbiota plays a critical role in maintaining healthy aging condition [8]. A study of extremely old individuals showed an increased abundance of *Christensenella*, *Akkermansia*, and *Bifidobacterium* in the gut of experimenters, suggesting a potential impact of gut microbes on life extension [9]. Furthermore, the gut microbiome of centenarians contains microorganisms carrying genes essential for secondary bile acid metabolism that imparts antimicrobial activity against Gram-positive pathogens, underlining the role of gut microbes in reducing infections in centenarians [10]. Collectively, the dynamic and modifiable nature of the gut microbiome provides potential therapeutic targets to tailor intervention strategies to address the health challenges associated with aging [11].

Perfluorobutanesulfonate (PFBS), a 4-carbon chain (C4) compound of full fluorination, is considered a safer alternative to perfluorooctanesulfonate (PFOS) because of a much shorter serum elimination half-life (25.8 days) than PFOS (4.8 years) [12]. However, the intensive use of PFBS and the transformation of its precursors inevitably cause environmental pollution [13]. Typically, in a survey of 79 cities in China, the median PFBS concentration in drinking water (1.21 ng/L) was consistently higher than those of PFOS (0.25 ng/L) and perfluorooctanoic acid (PFOA, 0.74 ng/L) [14]. Between 2009 and 2017, a significant 24-fold increase in PFBS concentration from 0.872 to 21.2 µg/L was noted in the groundwater near a fluorochemical industrial park in China [15]. Via multiple exposure routes (e.g., drinking water, food, and respiration), animals and humans are increasingly exposed to PFBS, as exemplified by the detection of PFBS in >95% of samples from Chinese women [16]. After entry into the organisms, PFBS-like persistent toxic contaminants cause dramatic fluctuations in the stability and resilience of the intestinal microbiota, thereby compromising the physiological health of the hosts [17,18]. It is a harsh fact that exposure to the detrimental effects caused by PFBS pollution may be implicated in the aging processes and further contribute to the onset and progression of age-related diseases [19]. Accumulating evidence has demonstrated that exposure to environmentally relevant concentrations of PFBS (100 μg/L) for 14 days dramatically disrupted the glucose and lipid metabolism as well as impacted the visual function in aged zebrafish [20,21,22]. Furthermore, PFBS also caused potent disturbances in sex endocrine homeostasis and follicular development in the aged [23].

In the context of the increasing sufferings from environmental pollution and multiple diseases, strategies to reestablish a normal gut by transferring feces from healthy individuals to the diseased recipient gut are being embraced in the field of healthy aging, including the treatment of inflammatory bowel disease, obesity, and diabetes [24,25,26]. Furthermore, our previous study found that transferring fecal microbiota from young donors into aged zebrafish recipients significantly refreshed the reproductive endocrine system and effectively ameliorated the adverse effects of PFBS [23]. However, the underlying mechanisms about the antagonistic action of young fecal microbiota against the reproductive endocrine disruptive toxicity of PFBS remain largely unknown. In this follow-up study, aged zebrafish (*Danio rerio*) were transplanted with feces from the young and then exposed to PFBS for 14 days. High-throughput transcriptomic sequencing was used here as a powerful tool to screen differentially expressed gene (DEG) profiles under the single or dual stress of young fecal transplants and PFBS. With the incorporation of various bioinformatics analyses, disturbances in biological pathways closely related to the functions of gonads were mapped to reveal the mechanisms of interaction between young fecal transplants and PFBS on an aged reproductive system.

## 2. Materials and Methods

### 2.1. Chemicals

PFBS was purchased from Tokyo Chemical Industry (purity >98%; CAS: 375-73-5). A stock solution of PFBS (10^5^ mg/L) was prepared in dimethyl sulfoxide (DMSO; Sigma-Aldrich, St. Louis, MO, USA). The other chemicals used in this study were of analytical grade.

### 2.2. Fish Maintenance and Exposure

**Figure 1:**
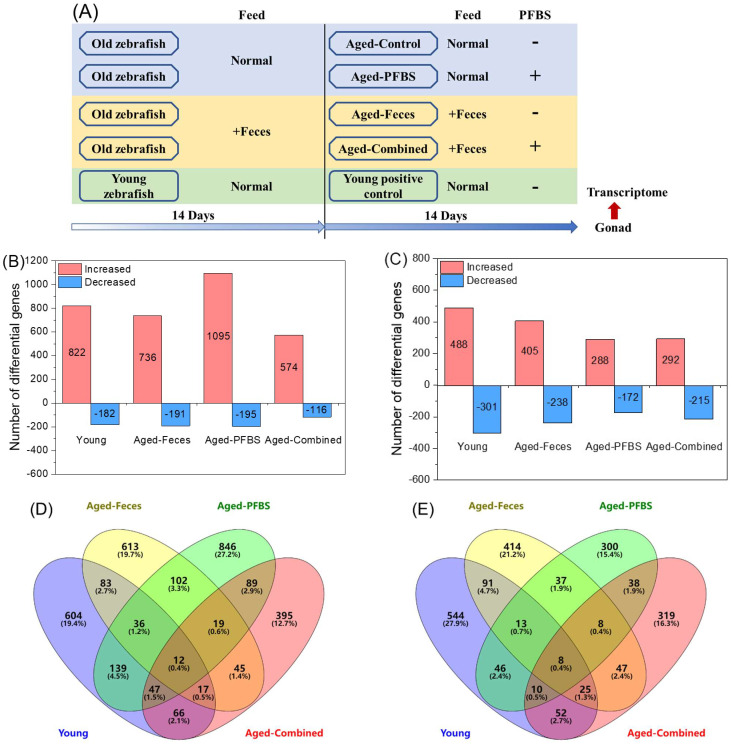
Experimental design (**A**) and a summary of the differential genes in the gonads of zebrafish after exposure to PFBS (0 and 100 μg/L) with or without the transplantation of young donor feces. The number of differential genes of significantly increased or decreased abundance in male (**B**) and female (**C**) zebrafish. A Venn diagram showing the common or unique genes among exposure groups in the male (**D**) and female (**E**). Values are presented as means of three replicates (*n* = 3). -, without; +, with. The two different shapes of the boxes indicate old and young fish, respectively (**A**).

Young zebrafish of 4 months of age and old zebrafish of 3 years of age were cultured in a semi-static system at 28 ± 0.5 °C on a 14/10 h day/night cycle. At the end of the two weeks of domestication, the aged zebrafish were fed the food supplemented with 5% *w*/*w* of freeze-dried feces from young donors for two weeks. Afterwards, the aged fish were exposed to 100 μg/L of PFBS for another two weeks, along with a continuous administration of young feces. The exposure dose of PFBS was selected according to previously reported worst-case concentrations in water samples (21.2 μg/L) [15], as well as the dose that was capable of impairing the reproductive endocrine balance in aged zebrafish (100 μg/L) [23]. The experiment consisted of four groups of the aged (aged-control group, aged-feces group, aged-PFBS group, and aged-combined group) and a positive control group of the young counterparts. A scheme of experimental design is presented in Figure 1A. Each group consisted of three replicate tanks (*n* = 3), and 20 males and 20 females were randomly assigned to 20 L of medium per tank. All tanks received an equal amount of DMSO (<0.001% *v*/*v*). The exposure solutions were changed daily to maintain proper water quality and chemical concentrations. After exposure, zebrafish were anesthetized in 0.03% MS222 (Sigma-Aldrich) and dissected on ice to obtain the gonads, which were then snap-frozen in liquid nitrogen and stored at −80 °C for subsequent transcriptional analysis. All the animal procedures were approved by the Animal Research and Ethics Committee of the Institute of Hydrobiology of the Chinese Academy of Sciences (Approval ID: IHB/ll/2022051).

### 2.3. High-Throughput Transcriptomic Sequencing

#### 2.3.1. Total RNA Isolation and Illumina Sequencing

The gonads of five random females or males in each tank were pooled together as one replicate, and each exposure group included three replicates (*n* = 3 per group). Total RNA was extracted from the testes and ovaries using Trizol^®^ reagent (Invitrogen, Carlsbad, CA, USA). After constructing the cDNA libraries, the Agilent 2100 bioanalyzer (Agilent Technologies, Palo Alto, CA, USA) and qRT-PCR were used to check the insert size and effective concentrations of the libraries. Then, the library preparations were sequenced on the Illumina Novaseq platform and 150 bp paired-end reads were generated.

#### 2.3.2. Bioinformatics Analyses

The raw data obtained from sequencing were filtered to remove reads with sequencing junctions and those with low sequencing quality to ensure the credibility of the following data analyses. Indexes of the *D. rerio* reference genome were constructed using HISAT2 v2.0.5, and paired-end clean reads were compared to the reference genome. The thresholds for DEGs in the four groups (aged-feces with transplantation of young feces, aged-PFBS with PFBS exposure, aged-combined with both transplantation of young feces and PFBS exposure, and positive control young zebrafish) relative to the aged-control group were set at *p* < 0.05 and |log2 (fold change)| > 1. Common and unique genes among exposure groups were shown by Venn diagrams, and the direction and magnitude of changes in DEGs were compared by heat maps. Principal component analysis (PCA) was performed by PAST software based on the variance–covariance matrix of DEGs for each treatment group [27]. Gene Ontology (GO) enrichment analysis of DEGs was implemented, and statistical enrichment of DEGs in the Kyoto Encyclopedia of Genes and Genomes (KEGG) pathway was analyzed using the cluster profile R package [28]. Protein–Protein Interaction (PPI) analysis of DEGs was performed by extracting the list of target genes from the STRING database to construct the network. Three models (D1, D2, and D3) simultaneously produced subnetworks, while only model D1 was considered in this study, which allowed no missing genes [29].

### 2.4. Quantitative Real-Time PCR Assays (qRT-PCR)

Alterations in gene transcriptions were determined by using qRT-PCR assays according to previous protocols [23]. Gonads from five zebrafish of the same sex were pooled together as one replicate (*n* = 3). Total RNA from gonads was extracted using Trizol^®^ reagent (Invitrogen) and reversely transcribed to cDNA with 1 μg of total RNA. Reverse transcription steps were performed according to the instructions of the commercial kit (Yeasen Biotech Co., Ltd., Shanghai, China). The transcriptional assay and analysis of target genes were performed by SYBR GreenKits (Yeasen Biotech Co., Ltd.) and CFX 384 Touch Real-Time PCR (Bio-Rad, Munich, Germany), respectively. Primer sequences for the genes were designed using the online Primer 3 software (http://frodo.wi.mit.edu/) (accessed on 10 May 2022) (Table S1). Given the transcriptional stability, ribosomal protein L8 (*rpl8*) was used as the reference gene and the relative gene transcript levels were calculated according to the 2^−ΔΔCT^ method.

### 2.5. Statistical Analyses

The values in this study are expressed as mean ± standard deviation (SD). The Shapiro–Wilk test and Levene’s test were used to verify the normality and homogeneity of the variances, respectively. Differences between groups were determined using a one-way analysis of variance (ANOVA) followed by the post hoc Tukey multiple comparison. Pearson’s linear fit and correlation methods were used to verify the consistency between RNA-seq and qPCR results. SPSS v22.0 (IBM Corporation, Armonk, NY, USA) and Origin 2022 (OriginLab Corporation, Northampton, MA, USA) were used for statistical analyses and image generation, respectively. Differences were considered statistically significant when *p* < 0.05.

## 3. Results and Discussion

### 3.1. Transcriptomic Sequencing Quality

Transcriptome sequencing yielded a total of 1,337,653,074 raw reads (664,826,490 and 672,826,584 reads from ovary and testis, respectively). After eliminating primer and adapter sequences and filtering out low-quality reads, Illumina deep sequencing of the ovary and testis left approximately 6 G of clean bases each, with a 94% efficiency rate (Tables S2 and S3).

### 3.2. Summary of DEGs

The DEGs were filtered and summarized relative to the aged-control group (Figure 1). Compared to the control, most testicular DEGs were significantly upregulated in the four exposure groups, especially in the aged-PFBS group (i.e., 1095 genes upregulated and 195 genes downregulated) (Figure 1B), supporting that PFBS could drastically disrupt the transcriptional activity in aged testes [23]. In contrast, the aged-combined group found the fewest changes in gene transcriptions with only 574 upregulated and 116 downregulated (Figure 1B), thus providing preliminary evidence that fecal transplantation from young donors mitigated the transcriptomic toxicity of PFBS in aging testis [23]. In addition, a distinct pattern of the transcriptome was noted in the gonads between the young and the elderly in characteristics of the large number of DEGs (Figure 1B,C). This is not surprising, because aging will result in the systemic functional turnover of multiple tissues [30]. The Venn diagram demonstrated that each group possessed a large proportion of unique genes (Figure 1D,E), pointing to the differential mechanisms of action.

**Figure 2:**
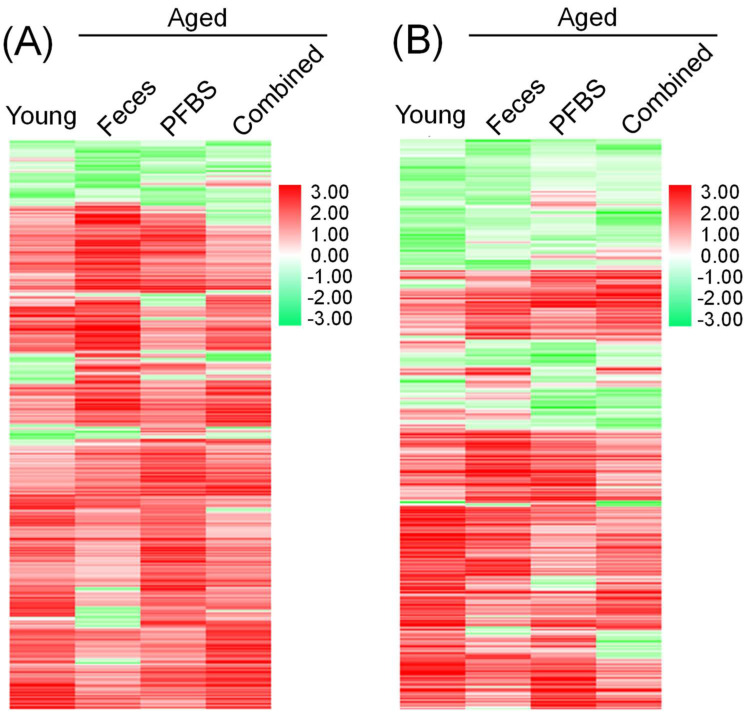
A heatmap showing the overall changes in differential genes among exposure groups in male (**A**) and female (**B**) zebrafish. Red and green colors stand for increased and decreased changes, respectively, compared to the aged−control abundance. Color intensity is proportional to the changing magnitude. Values are presented as means of three replicates (*n* = 3).

**Figure 3:**
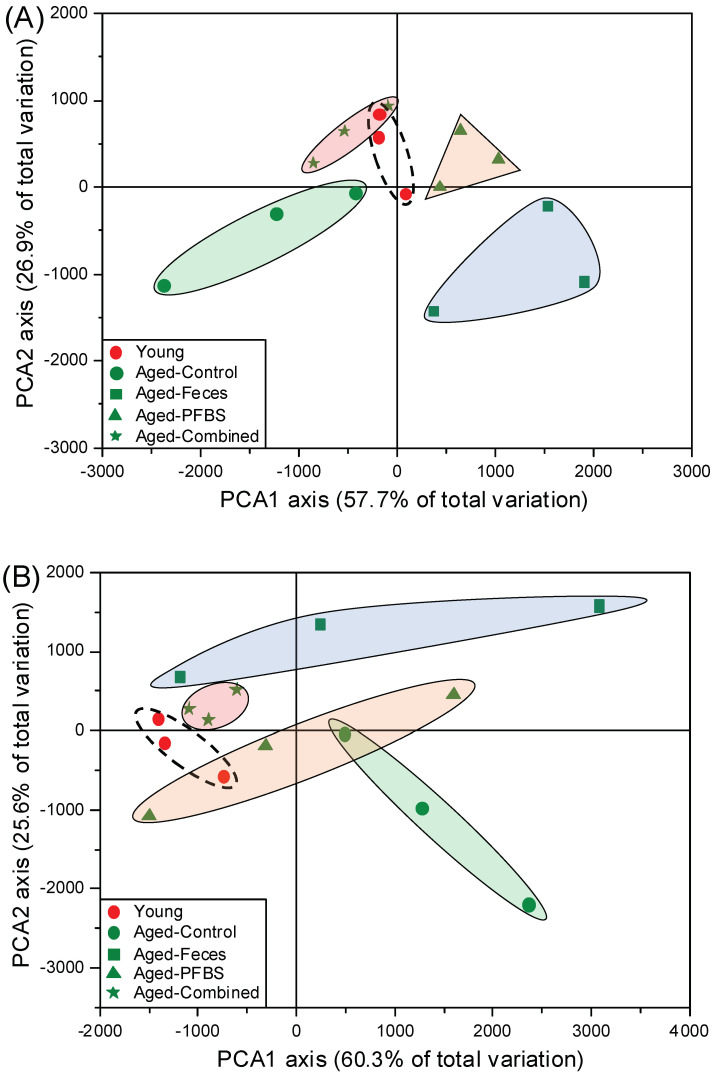
Principal component analysis (PCA) in male (**A**) and female (**B**) zebrafish, with the input of differential genes based on the variance–covariance matrix.

Heat map clustering further confirmed the unique distribution of genes in each group with regard to the changes in trend and magnitude (Figure 2). The PCA plots grouped replicate samples along the x and y axes according to the list of DEGs (Figure 3). All the four groups including young, aged-feces, aged-PFBS, and aged-combined groups were separated away from the aged-control group in both males and females, indicating the variation in transcriptomic fingerprint as functions of age, young feces transplantation, and PFBS. It was notable that sample clusters from the young and aged-combined groups were overlapped to certain degree (Figure 3A,B), further highlighting that, regardless of PFBS intoxication, young fecal transplantation efficiently refreshed the reproductive activities and formed a young-like phenotype of the gonadal transcriptome in the aged recipients [23]. Consistent with the present findings, a fecal microbiota transplant from young mice donors was previously found to transform the gut microbial community of aged mice into a youth-like ecosystem [31].

### 3.3. GO, KEGG, and PPI Analyses Based on DEGs

**Figure 4:**
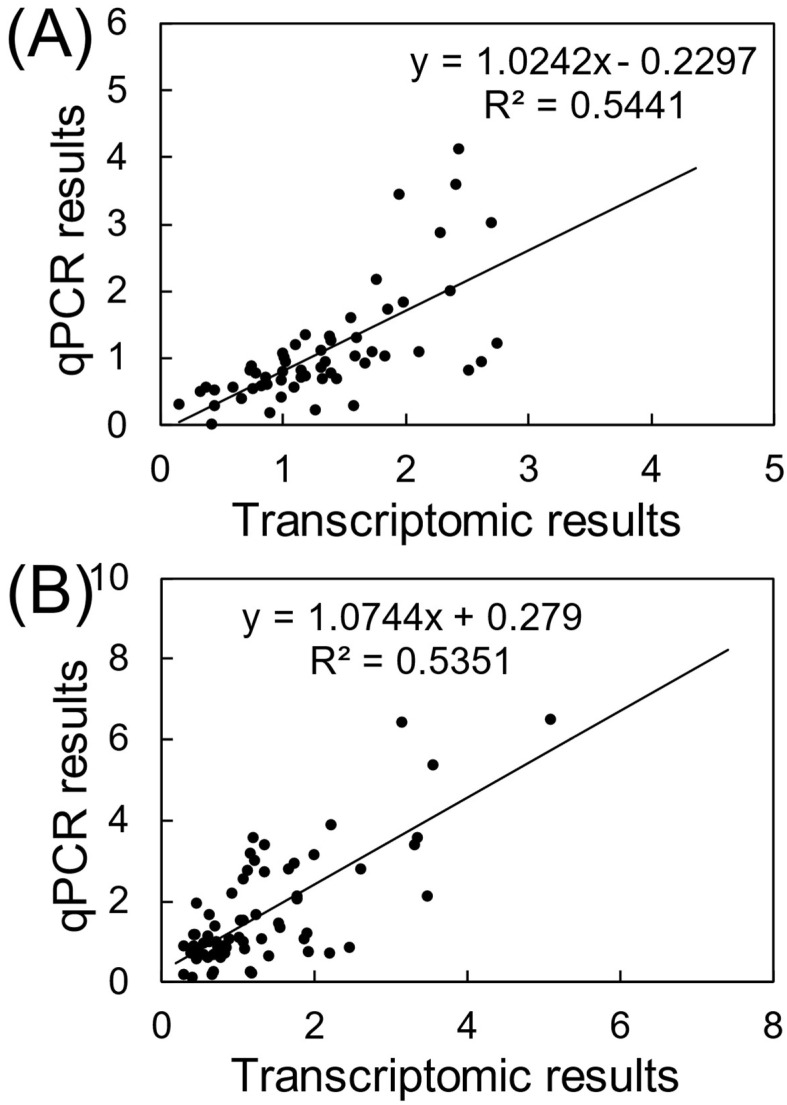
Correlation analysis between transcriptomic and qPCR results in male (**A**) and female (**B**) zebrafish.

**Figure 5:**
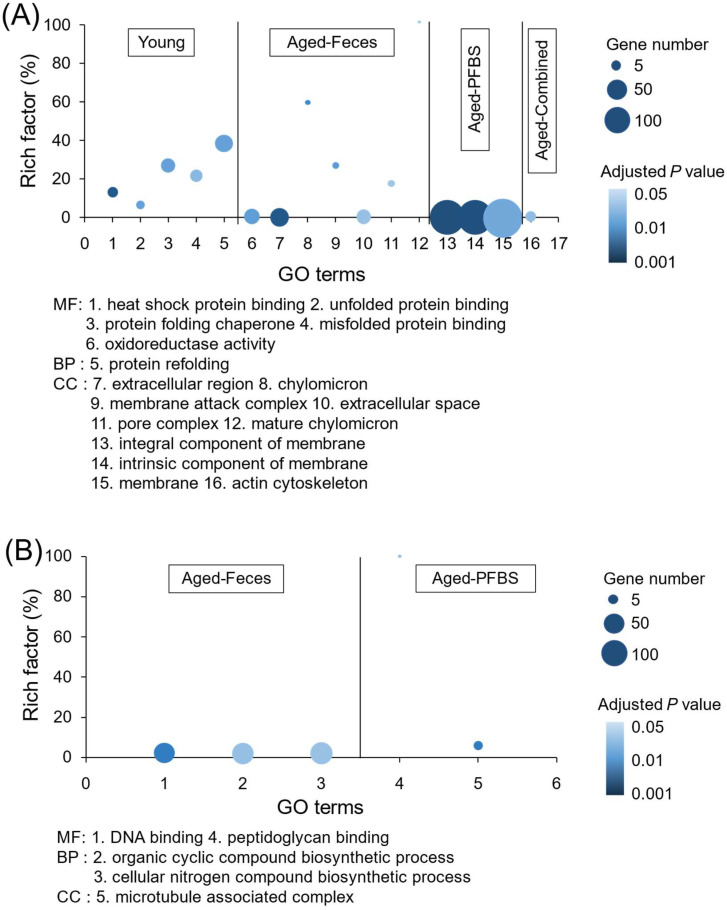
Significantly enriched GO terms (MF, molecular function; BP, biological process; CC, cellular component) in male (**A**) and female (**B**) zebrafish gonads after exposure to PFBS (0 and 100 μg/L) with or without the transplantation of young donor feces. Color intensity is proportional to the enrichment significance (adjusted *p* value). The circular area represents the number of inclusive genes.

**Figure 6:**
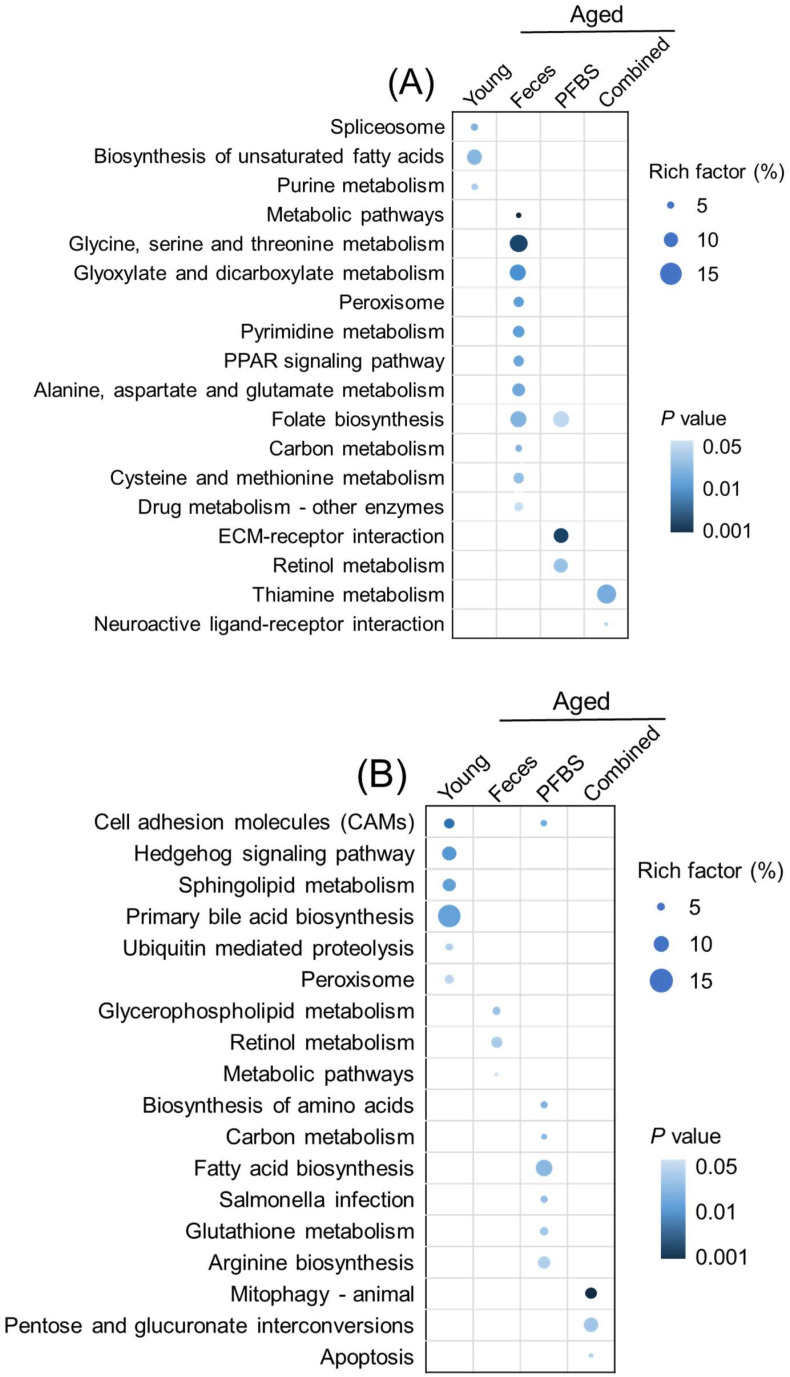
KEGG pathways of significant enrichment in male (**A**) and female (**B**) zebrafish gonads after exposure to PFBS (0 and 100 μg/L) with or without the transplantation of young donor feces. Color intensity is proportional to the enrichment significance (*p* value). The circular area represents the rich factor.

Transcriptions of representative DEGs were also validated by using qRT-PCR assays (Table S4). There was a good consistency of gene transcriptional changes between qRT-PCR and RNA-seq assays, verifying the reliability of the transcriptomics analyses (Figure 4). By uploading the list of DEGs, alterations in biological functions in gonads were identified based on a suite of bioinformatics analyses. During aging, changes in the composition and function of gut microbiota trigger a decline in normal intestinal epithelial barrier function, which, in turn, fundamentally disrupts the balance of host physiological health and metabolism activities [32]. In the present study, a complex gene interactive network was established for the young males relative to the aged control, verifying the comprehensive distinction between the young and the elderly in system biology (Figure S1). Furthermore, among the GO terms of young males, the most representative biological process was integrated as protein refolding, with particular molecular function involving protein synthesis, such as heat shock protein (HSP) binding, unfolded protein binding, protein folding chaperone, and misfolded protein binding (Figure 5A). The outstanding metabolic capacity of the young allows ready maintenance of the stability of the correct protein topography and degrades the unwanted or misfolded proteins [33]. However, in aging and age-related diseases, the imbalance in protein homeostasis leads to the accumulation of misfolded proteins, eventually inducing cellular dysfunction and apoptosis [34]. In addition to contributing to the folding and function of fragile or misfold-prone proteins, HSPs have an important role in the regulation of cellular redox homeostasis [35]. It is well known that persistent redox imbalance inevitably leads to further aging and related diseases [36]. Consistently, biosynthesis of unsaturated fatty acids was the main KEGG pathway for the enrichment of DEGs in young males (Figure 6A). Accumulative findings have suggested that increasing unsaturated fatty acid biosynthesis or preventing its catabolism can improve animal models’ metabolic health and longevity [37]. Interestingly, the KEGG pathway in young females was enriched with more pathways, mainly including lipid metabolism (i.e., primary bile acid biosynthesis, sphingolipid metabolism, and peroxisome) as well as cell proliferation, differentiation, and migration (e.g., cell adhesion molecules (CAMs) and hedgehog signaling pathway) (Figure 6B). A strong link between aging and alterations in the ovarian microenvironment has been demonstrated [38]. A larger number of follicles and fewer apoptotic cells are generally found in young mice compared to the aged [31].

The present study further found that substantial changes in aging gonads were caused by young fecal transplantation or/and PFBS exposure. Previous studies have highlighted the efficacy of fecal transplants from young donors on shaping the developmental rhythm of gametes in the gonads of aged zebrafish recipients [23]. Based on DEGs, the PPI analysis showed that young fecal transplants typically affected the retinol metabolism pathway in both sexes (Figure S1B,C). The KEGG enrichment pathway demonstrated that transplantation of feces from the young altered various metabolic pathways in the elderly (Figure 6), thereby validating the therapeutic potential of manipulating gut microbes to enhance systemic metabolism and then restore healthy aging. Consistently, a recent study has shown that fecal transplantation could reverse the high bone turnover of senile osteoporosis and alleviate aging-related bone loss [39]. In addition, compared to aged females, young fecal transplants were able to re-shape more metabolic pathways in aged males, including glyoxylate and dicarboxylate metabolism; glycine, serine, and threonine metabolism; pyrimidine metabolism; alanine, aspartate, and glutamate metabolism; cysteine and methionine metabolism; drug metabolism (Figure 6). The symbiotic gut microbiota is critical in the production and regulation of sex hormones, interacting in a concordant manner via the estrobolome [40]. A previous study reported that the enhanced steroid hormone biosynthesis induced by young fecal transplants increased the release of sex hormones [41]. The inherent differences in sex hormone levels between males and females, in turn, confer differences in the composition and abundance of gut microbes [42], which may explain the sex-dependent differences in the efficacy of gut microbiota-based manipulation measures.

PFBS exposure is capable of disrupting gamete differentiation, proliferation, and maturation in the gonads, consequently resulting in reproductive toxicity [43]. Specifically, PFBS single exposure of the aged males in this study had apparent effects on biological functions relevant to cell development, including the integral component of the membrane and intrinsic component of the membrane (Figure 5A). The predominantly enriched KEGG pathways (i.e., ECM–receptor interaction, retinol metabolism, and folate metabolism) further emphasized the disruption of cell proliferation by PFBS in the aged male (Figure 6A). Mechanistically, the ECM–receptor interaction signaling pathway can affect germ cell migration by enhancing cell adhesion [44]. An important bioactive metabolite along the retinol metabolism pathway is retinoic acid (RA), which is deemed an essential regulatory compound in meiosis during spermatogenesis [45]. Disturbances in RA synthesis kinetics may disrupt the spermatogenic process, thus affecting the reproductive success. Previous studies have confirmed that PFBS exposure significantly depleted the spermatogonia reserve in the aged testis [23]. In addition, the B vitamin folic acid is also required by various metabolic functions, such as DNA replication, repair, and methylation, carbohydrate metabolism, and synthesis of nucleotides and vitamins [46]. In contrast, toxic action of PFBS in aged ovaries was mainly implicated in the microtubule-associated complex and intervention with fatty acid biosynthesis and arginine biosynthesis signaling pathways (Figure 5B and Figure 6B). It is known that disruption of the microtubule-based cytoskeleton will lead to the shedding of germ cells [47]. As a component of lipids, fatty acids are key components of all biological membrane structures, while abnormal fatty acid synthesis may disorganize the membrane structural integrity and expansion during cell proliferation and division [48]. Similarly, our previous study confirmed that oocyte development was blocked by PFBS in the aging female ovary, thereby reducing egg growth and production [23].

**Figure 7:**
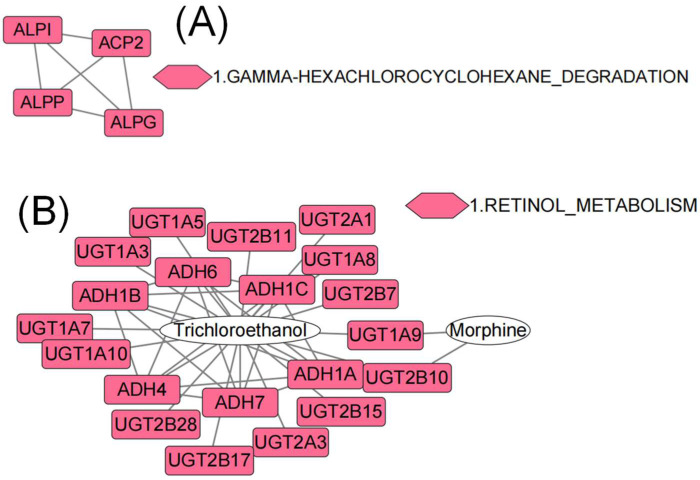
The most significant gene interactive network for the aged-combined group in male (**A**) and female (**B**) according to R spider enrichment of differential genes. The colored node is labeled by the human homologs of differential genes, where the same color represents the enrichment along the same pathway. Only overrepresented pathways are colored. The number preceding the pathway denotes the hierarchy order.

It was intriguing to note that the co-exposure paradigm had a milder influence on the aged than PFBS, further confirming the efficacy of young fecal transplants to mitigate the hazardous effects of environmental xenobiotics. Various biological processes that are related to reproduction and testicular development were significantly modulated by the combined exposure, including the cellular actin cytoskeleton (Figure 5A), thiamine metabolism for energy, neuroactive ligand–receptor interaction (Figure 6A), and gamma hexachlorocyclohexane degradation (Figure 7A). A different set of pathways were significantly enriched in aged females from the combined group, comprising pentose and glucuronate interconversions, mitophagy, and apoptosis (Figure 6B). It is recognized that mitophagy and apoptosis can promote longevity in part by removing defective mitochondria or cells that accumulate during the aging process [49]. In addition, PPI network construction verified a profound effect of co-exposure on retinol metabolism in aged ovaries (Figure 7B). Retinol is documented to possess an anti-stress effect on the ovary and subsequently contribute to follicle cell proliferation and development [50]. The intake of moderate amounts of retinol can aid ovarian recovery and significantly improve reproductive efficiency [51].

## 4. Conclusions

The potential efficacy of young fecal transplantation in zebrafish gonads against the dual stress of aging and PFBS was explored by high-throughput transcriptomics. The present study highlighted that the transplantation of young feces efficiently activated various physiological processes and enhanced metabolism capacity in the elderly. Furthermore, transferring young fecal microbiota to the aged antagonized the apparent inhibition of cell proliferation in the gonads caused by PFBS, providing mechanistic clues about the capability of gut microbial remediation against PFBS reproductive toxicity. Notably, the varying efficacy of young fecal transplants depending on sex pointed to a sex specificity. Future work is necessary to elucidate the crosstalk between fecal transplantation and the PFBS pollutant on reproductive and transgenerational toxicity under aging challenge.

## Data Availability

Not applicable.

## Supplementary Materials

The following are available online at https://www.mdpi.com/article/10.3390/toxics10110631/s1, Figure S1: The most significant gene interactive network for young and aged feces groups in males, and aged feces group in females, Table S1: Primers of the selected genes for qRT-PCR assay, Table S2: Summary of transcriptomic sequencing data and quality in male zebrafish, Table S3: Summary of transcriptomic sequencing data and quality in female zebrafish, Table S4: The qRT-PCR results [52,53,54,55,56,57].
