# Supplementary material for: Transcriptomic Interaction between Young Fecal Transplantation and Perfluorobutanesulfonate in Aged Zebrafish Gonads
Source: Toxics. 2022 Oct 22;10(11):631. doi: 10.3390/toxics10110631 (PMC9692687; doi:10.3390/toxics10110631)
Supplement: Supplementary file 1 [file toxics-10-00631-s001.zip › toxics-1948850-supplementary.pdf]

toxics

MDPI

Supplementary Materials: Transcriptomic Interaction between Young Fecal Transplantation and Perfluorobutanesulfonate in Aged Zebrafish Gonads

Lizhu Tang, Jing Li, Baili Sun, Yachen Bai, Xiangzhen Zhou and Lianguo Chen

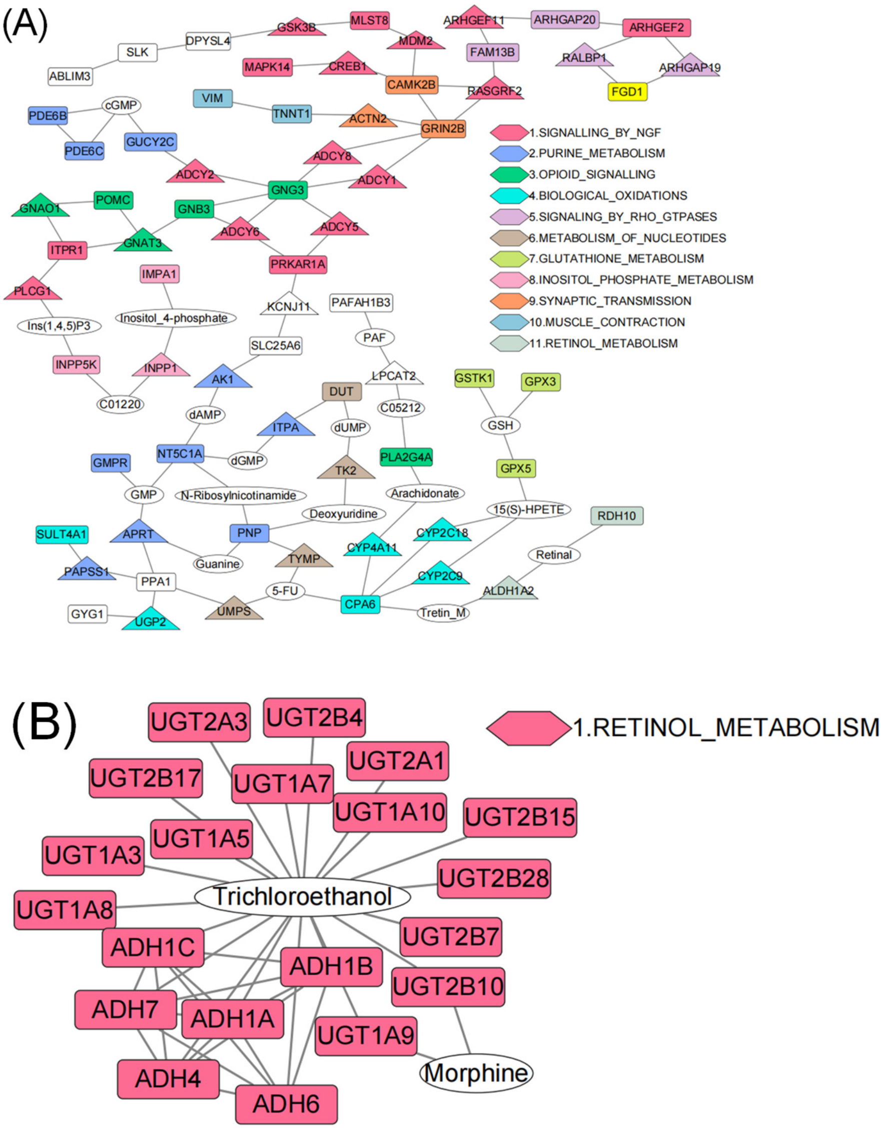

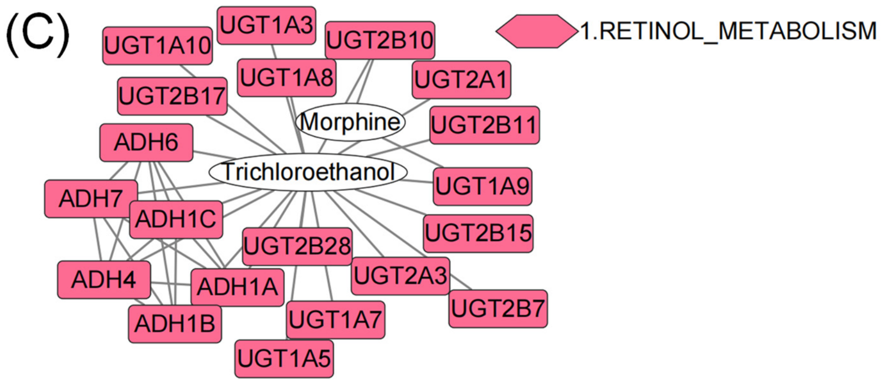

Figure S1. The most significant gene interactive network for young (A) and aged feces (B) groups in males, and aged feces group in females (C) based on R spider enrichment of differential genes. The colored node is labeled by the human homologs of differential genes, where the same color represented enrichment into the same pathway. Only overrepresented pathways are colored. The number preceding the pathway denotes the hierarchy order.

Table S1. Primers of the selected genes for qRT-PCR assay.

Genes	Sequence of primers (5'–3')	Reference	Accession number	Amplification efficiency
acvr2ab	F: CCATGAGAAGGGTTCACTGAC<br>R: TAGATCACTCTTCAGCAGCAC	Dogra et al.,2017	XM_005168700.4	99.2%
igf3	F: GCCAAACGCCTTCAGATAATGC<br>R: GCTGCTCCAGGTTTGCCTATGT	Li et al., 2018	NM_001115050.1	99.0%
inhbaa	F: TGCTGCAAGCGACAATTTTA<br>R: CATTCGTTTCGGGACTCAAG	Wang and Ge, 2004	NM_001362254.1	93.9%
insb	F: CCTGGAGACCTTGCTGGCTT<br>R: CCAGGTGGTAGATGGTGCAGG	Lou et al., 2020	NM_001039064.1	93.2%
ptgs2a	F: TTATGGAGAGACGCTGGAGGT<br>R: GATGAGACGAGTGGTTTGGAA	Tang et al., 2016	NM_153657.1	99.3%
rpl8	F: TTGTTGGTGTTGTTGCTGGT<br>R: GGATGCTCAACAGGGTTCAT	Chen et al., 2012	NM_200713.1	99.6%

References

Chen, L.; Yu, K.; Huang, C.; Yu, L.; Zhu, B.; Lam, P.K.S.; Lam, J.C.W.; Zhou, B. Prenatal Transfer of Polybrominated Diphenyl Ethers (PBDEs) Results in Developmental Neurotoxicity in Zebrafish Larvae. *Environ. Sci. Technol.* **2012**, *46*, 9727–9734. https://doi.org/10.1021/es302119g.

Dogra, D.; Ahuja, S.; Kim, H.; Rasouli, S.; Stainier, D.; Reischauer, S. Opposite Effects of Activin Type 2 Receptor Ligands on Cardiomyocyte Proliferation during Development and Repair. *Nat. Commun.* **2017**, *8*, 1902. https://doi.org/10.1038/s41467-017-01950-1.

Li, J.; Niu, C.; Cheng, C. Igf3 Serves as A Mediator of Luteinizing Hormone in Zebrafish Ovulation. *Biol. Reprod.* **2018**, *6*, 99. https://doi.org/10.1093/biolre/ioy143.

Lou, B.; Boger, M.; Bennewitz, K.; Sticht, C.; Kopf, S.; Morgenstern, J.; Fleming, T.; Hell, R.; Yuan, Z.; Nawroth, P.; Kroll, J. Elevated 4-Hydroxynonenal Induces Hyperglycaemia via Aldh3a1 Loss in Zebrafish and Associates with Diabetes Progres-sion in Humans. *Redox Biol.* **2020**, *37*, 101723. https://doi.org/10.1016/j.redox.2020.101723.

Tang, H.; Liu, Y.; Li, J.; Yin, Y.; Li, G.; Chen, Y.; Li, S.; Zhang, Y.; Lin, H.; Liu, X.; Cheng, C.H.K. Gene Knockout of Nuclear Progesterone Receptor Provides Insights into the Regulation of Ovulation by LH Signaling in Zebrafish. *Sci. Rep.* **2016**, *6*, 28545. https://doi.org/10.1038/srep28545.

Wang, Y.; Ge, W. Developmental Profiles of Activin βA, βB, and Follistatin Expression in the Zebrafish Ovary: Evidence for Their Differential Roles during Sexual Maturation and Ovulatory Cycle. *Biol. Reprod.* **2004**, *71*, 6. https://doi.org/10.1095/biolreprod.104.032649.

Table S2. Summary of transcriptomic sequencing data and quality in **male** zebrafish.

Samples	Raw Base (G)	Clean Base (G)	Raw Reads	Clean Reads	Effective Rate (%)	Error Rate (%)	Q20 (%)	Q30 (%)	GC Content (%)
Young-1	6.34	5.88	42,265,414	39,226,198	92.81	0.03	97.48	93.44	42.79
Young-2	6.82	6.42	45,442,946	42,804,876	94.19	0.03	97.54	93.58	44.29
Young-3	6.64	6.29	44,244,646	41,906,240	94.71	0.03	97.59	93.63	44.82
Aged Control-1	6.6	6.31	44,005,202	42,060,958	95.58	0.03	97.69	93.69	40.54
Aged Control-2	6.47	6.17	43,163,778	41,140,732	95.31	0.03	97.82	94.09	45.21
Aged Control-3	7.27	6.63	48,443,266	44,187,158	91.21	0.03	97.67	93.85	44.62
Aged Feces-1	6.83	6.42	45,512,514	42,790,570	94.02	0.03	97.62	93.64	41.42
Aged Feces-2	6.79	6.48	45,257,058	43,175,266	95.40	0.03	97.78	94.05	43.3
Aged Feces-3	6.42	6.05	42,825,706	40,334,516	94.18	0.03	97.71	93.9	44.82
Aged PFBS-1	6.88	6.24	45,840,308	41,587,194	90.72	0.03	97.57	93.63	42.06
Aged PFBS-2	6.89	6.44	45,902,918	42,931,564	93.53	0.02	98.05	94.62	44.68
Aged PFBS-3	6.85	6.67	45,675,528	44,460,020	97.34	0.03	97.74	93.73	43.82
Aged Combined-1	6.53	6.27	43,520,560	41,771,464	95.98	0.02	97.97	94.39	45.53
Aged Combined-2	6.65	6.37	44,316,666	42,477,396	95.85	0.03	97.82	94.07	45.24
Aged Combined-3	6.96	6.44	46,410,074	42,945,688	92.54	0.03	97.32	93.02	40.31

Table S3. Summary of transcriptomic sequencing data and quality in **female** zebrafish.

Samples	Raw Base (G)	Clean Base (G)	Raw Reads	Clean Reads	Effective Rate (%)	Error Rate (%)	Q20 (%)	Q30 (%)	GC Content (%)
Young-1	6.92	6.48	46,158,320	43,180,034	93.55	0.03	97.88	94.2	43.86
Young-2	6.51	5.96	43,369,826	39,755,112	91.67	0.03	97.91	94.26	43.16
Young-3	6.51	6.09	43,369,924	40,592,200	93.60	0.02	97.95	94.32	43.36
Aged Control-1	6.87	6.52	45,776,318	43,481,124	94.99	0.03	97.82	94.03	41.14
Aged Control-2	6.52	6.52	43,490,330	43,450,030	99.91	0.03	97.65	93.74	39.36
Aged Control-3	7.33	6.89	48,887,766	45,958,740	94.01	0.03	97.7	93.84	46.07
Aged Feces-1	6.52	6.01	43,452,702	40,083,762	92.25	0.02	97.95	94.3	43.45
Aged Feces-2	6.85	6.24	45,686,756	41,619,012	91.10	0.03	97.8	93.98	44.11
Aged Feces-3	6.83	6.38	45,509,290	42,537,478	93.47	0.03	97.79	93.95	42.35
Aged PFBS-1	6.89	6.44	45,966,090	42,930,132	93.40	0.03	97.78	93.92	42.96
Aged PFBS-2	6.38	5.95	42,551,228	39,634,662	93.15	0.03	97.75	93.92	42.32
Aged PFBS-3	5.85	5.84	38,996,610	38,960,592	99.91	0.03	97.62	93.63	38.85
Aged Combined-1	6.19	5.73	41,279,416	38,185,882	92.51	0.03	97.81	93.99	43.34
Aged Combined-2	6.63	6.23	44,203,544	41,540,026	93.97	0.03	97.66	93.58	41.8
Aged Combined-3	6.92	6.55	46,128,370	43,668,114	94.67	0.03	97.75	93.81	41.86

Table S4. The qRT-PCR results of differential genes of aged zebrafish after exposure to PFBS (0 and 100 μg/L) with or without young zebrafish feces.[a]

Genes		Young	Aged			
			Control	Feces	PFBS	Combined
Male	*acvr2ab*	2.8±0.89	1.02±0.27	6.41±1.44	4.3±0.40	2.94±0.12
	igf3	1.36±0.60	1.17±0.82	0.49±0.28	0.57±0.37	0.62±0.32
	inhbaa	0.63±0.20	1±0.10	0.69±0.33	0.68±0.27	0.59±0.23
	insb	0.81±0.17	1.09±0.53	1.82±1.36	1.55±0.34	0.64±0.35
	ptgs2α	2.71±0.42	1.04±0.41	2.69±2.01	1.15±0.55	0.54±0.26
Female	*acvr2ab*	0.61±0.33	1.03±0.27	1.44±0.98	1.05±0.13	2.07±0.59
	igf3	2.09±0.63	1.01±0.19	2.61±0.94	2.88±0.71	1.95±0.69
	inhbaa	2.76±1.97	1.04±0.33	3.79±1.59	2.51±0.36	2.48±1.16
	insb	0.21±0.06***	1.05±0.43	0.74±0.15	0.97±0.14	0.81±0.06
	ptgs2α	0.3±0.04	1.13±0.73	1.46±1.04	0.23±0.06	7.67±2.69

[a] Values represent the mean ± SD of three replicates (*n* = 3); ****P* < 0.001 indicates significant differences compared to the aged control group.
